# Supplementary material for: Electronic Paddlewheels Impact the Dynamics of Superionic Conduction in AgI
Source: Chemphyschem. 2025 Oct 16;26(21):e202500077. doi: 10.1002/cphc.202500077 (PMC12597220; doi:10.1002/cphc.202500077)
Supplement: Supplementary file 1 — Supplementary Material [file CPHC-26-e202500077-s001.pdf]

Supplementary Information for
"Electronic Paddlewheels Impact the Dynamics of Superionic Conduction in AgI"

Harender S. Dhattarwal[1] and Richard C. Remsing[1,*]

[1]*Department of Chemistry and Chemical Biology, Rutgers University, Piscataway, NJ 08854*

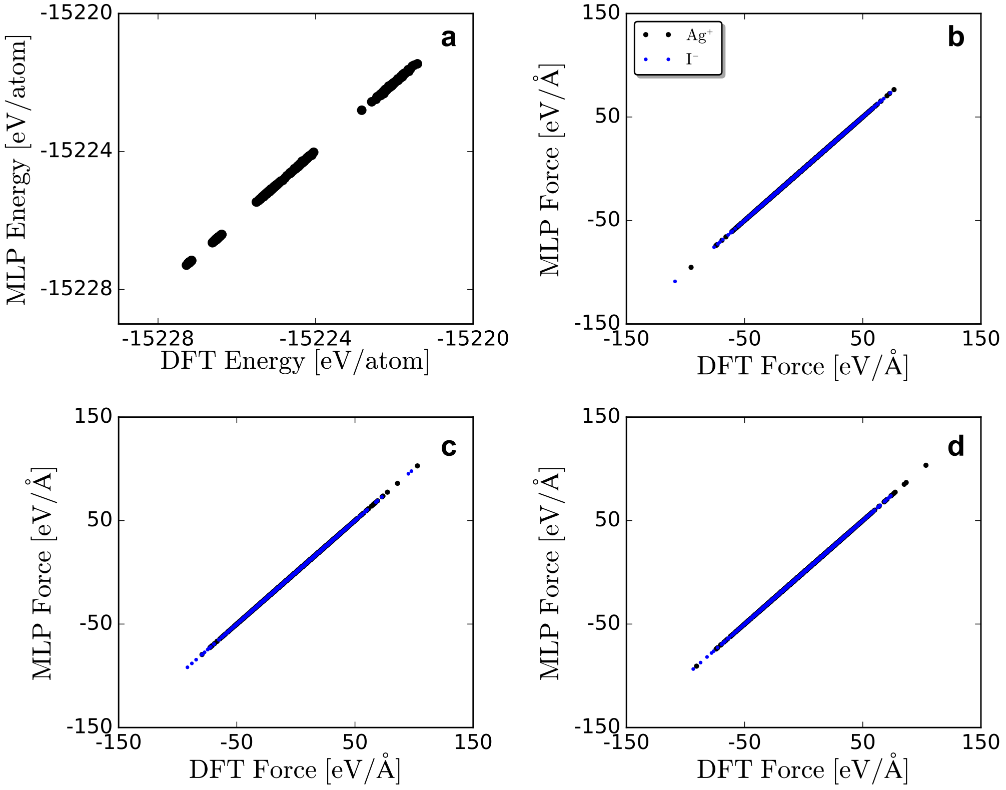

Figure S 1: Parity plot for the (a) total energy per atom of AgI systems, forces in (b) x, (c) y, and (d) z directions.

* rick.remsing@rutgers.edu

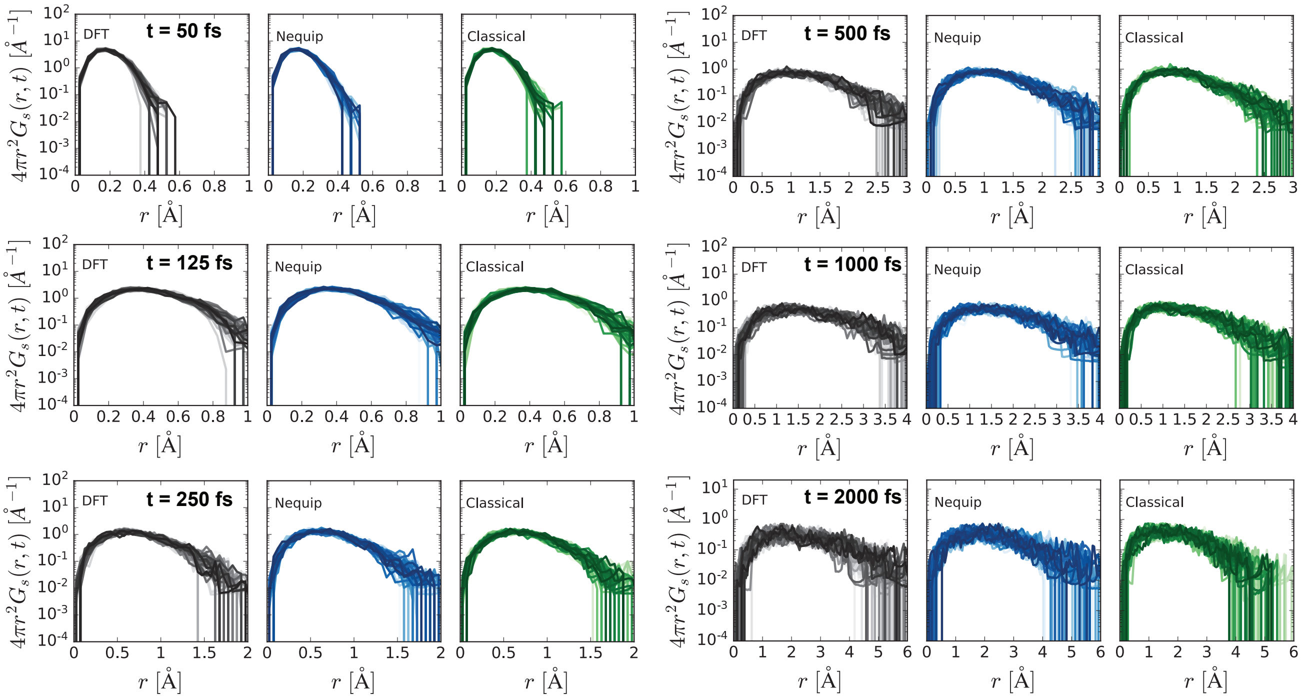

Figure S 2: Self-part of the van Hove correlation function, $G_s(r,t)$, for individual silver cations; each curve corresponds to a different ion. Different models are indicated in each figure (DFT, NequIP, Classical), and the time at which the correlation function is evaluated is indicated in the first (DFT) panel of each set of three. Note the change in scale of the x-axis at time increases.

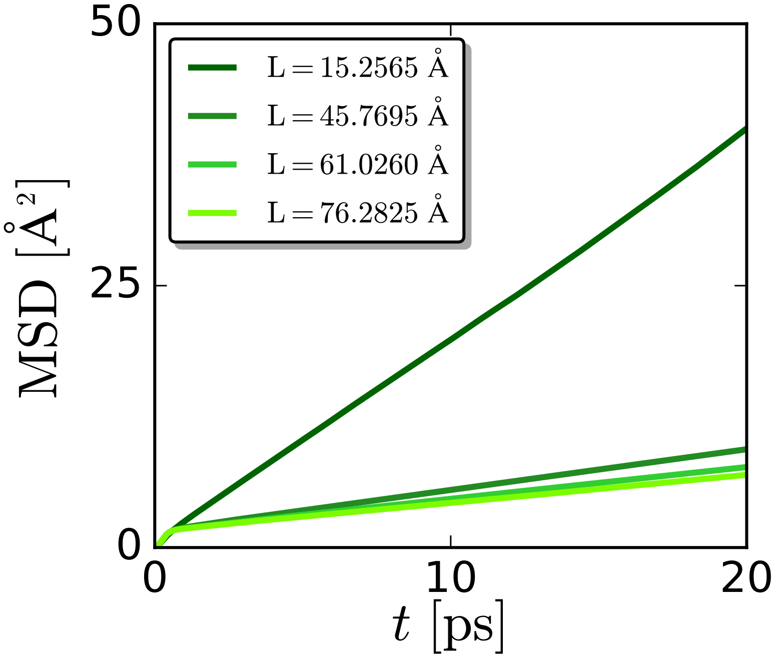

Figure S 3: Mean square displacement of Ag^+ for different system sizes obtained from FF models.

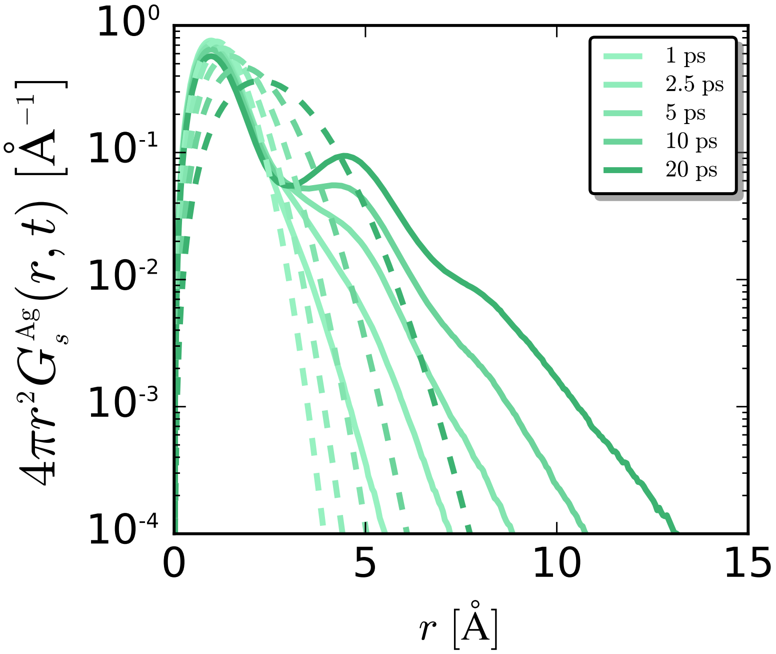

Figure S 4: Self-part of the van Hove correlation function, $G_s(r,t)$, for silver cations, calculated for AgI system with 6912 particles and box dimensions of 61.026 Å. Dashed lines correspond to Gaussian distributions with the same mean and variance as the correspond simulated $G_s(r,t)$ of the same color.
